# Steel Rack Connections: Identification of Most Influential Factors and a Comparison of Stiffness Design Methods

**DOI:** 10.1371/journal.pone.0139422

**Published:** 2015-10-09

**Authors:** S. N. R. Shah, N. H. Ramli Sulong, Mahdi Shariati, M. Z. Jumaat

**Affiliations:** Department of Civil Engineering, University of Malaya, 50603, Kuala Lumpur, Malaysia; China Medical University, TAIWAN

## Abstract

Steel pallet rack (SPR) beam-to-column connections (BCCs) are largely responsible to avoid the sway failure of frames in the down-aisle direction. The overall geometry of beam end connectors commercially used in SPR BCCs is different and does not allow a generalized analytic approach for all types of beam end connectors; however, identifying the effects of the configuration, profile and sizes of the connection components could be the suitable approach for the practical design engineers in order to predict the generalized behavior of any SPR BCC. This paper describes the experimental behavior of SPR BCCs tested using a double cantilever test set-up. Eight sets of specimens were identified based on the variation in column thickness, beam depth and number of tabs in the beam end connector in order to investigate the most influential factors affecting the connection performance. Four tests were repeatedly performed for each set to bring uniformity to the results taking the total number of tests to thirty-two. The moment-rotation (M-θ) behavior, load-strain relationship, major failure modes and the influence of selected parameters on connection performance were investigated. A comparative study to calculate the connection stiffness was carried out using the initial stiffness method, the slope to half-ultimate moment method and the equal area method. In order to find out the more appropriate method, the mean stiffness of all the tested connections and the variance in values of mean stiffness according to all three methods were calculated. The calculation of connection stiffness by means of the initial stiffness method is considered to overestimate the values when compared to the other two methods. The equal area method provided more consistent values of stiffness and lowest variance in the data set as compared to the other two methods.

## Introduction

During the last few decades, the growing number of industrial warehouses and supermarkets throughout the world has given significant rise to the importance of structures that could solve the storage capacity and goods handling problems in the storage buildings. Steel pallet racks (SPRs) are considered as the perfect storage solution and provide sufficient and readily accessible storage when less space is available compared to the high volume of storage items. These three-dimensional structures provide direct and easy access to all the stored items and are readily demountable and capable of reassembly.

The effective use of SPRs demands flexibility in the material constituting these racks. Thus, cold formed steel is preferred for the manufacturing of these peculiar structures in order to permit the handy adjustment and reassembling of rack elements upon requirement and due to its good strength-to-weight ratio [1]. However, AS4084 [2] permits the use of hot rolled steel for manufacturing when the rack has to support heavy loads.

A typical SPR has several structural members. Conventionally, the columns used in rack structures are made up of cold-formed steel; however, in some cases, more traditional hot-rolled profiles are used as well as tubular hollow sections. The thickness of these columns usually ranges between 1.5 mm and 3 mm, which is comparatively negligible to the sufficiently greater height of the column. Due to the high slenderness and perforations provided in the column, the critical elastic flexural and flexural-torsional (global) buckling loads are less than the same column without holes [3, 4]. Moreover, local buckling may occur, where the section involves plate flexure alone without transverse deformation of the overall column, or distortional buckling, where the cross-sectional shape changes along the length of the member without transverse deformation.

The beams used in these rack structures are normally box, hat or channel sections with sufficient bending capacity. Diagonal bracing is provided in cross-aisle direction. Bolted connections between cross-aisle bracing and columns are usually used in Australia and Europe, while manufacturers in the United States usually use welded connections. The other components are the beam-to-column connections (BCCs) and base connection, which are highly responsible for the stability and overall performance of rack structures due to the unavailability of bracing in the down-aisle direction in order to provide the consumer quick and unblocked access to the stored goods.

BCC in SPRs is mainly established by hook-in end connectors made up of hot rolled alloy steel. The important part of the beam end connector is the ‘tab’, which serves as a junction between the beam and column. The beam end connectors are engaged in column perforations with the help of a safety lock, which ensures that the end connector is correctly engaged to the column. The connector should have adequate strength to avoid sway failure of this portion. The beam end connectors are tightly attached to the columns through their tabs. This phenomenon may result in some initial looseness of the joint, which increases the bending moment similar to the bending moments caused by lateral loads. This increases the sway effect and shear force upon the strength of the whole structure.

The SPR BCC requires careful design consideration mainly due to the aberrant constructional design and individually variable behavior of the hooked connection used by rack manufacturers. Furthermore, a certain looseness and the relatively small rotational stiffness with regard to the customary connections in steel buildings, are some additional factors that need an accurate estimation of the connection’s behavior for design purposes. The BCCs in building structures are traditionally considered to fulfill the conditions of either a hinge or a fixed-end restraint. The SPR BCCs are treated as semi-rigid connections and the structural analysis is carried out by adopting a semi-continuous sway frame model [1].

The peculiar characteristics of SPR BCCs have been evaluated by several researchers through experimental and numerical investigations. Markazi et al. [5] provided a broad classification of commercially available beam end connectors and performed experimental investigations on SPR BCCs to determine the factors affecting the performance of the beam end connector through the cantilever test method. Several products were tested. The size of the members attached to the connector, particularly the column, the gauge of the beam end connector and the column, and the welding method of the beam and beam end connector highly influenced the performance of the connection. The rotation component of the column was not considered in the study of Markazi et al. [5]. Bernuzzi and Castiglioni [6] determined the effect of cyclic loading on the connection and the way it affects the overall rack performance. Kozlowski and Slęczka [7] performed experimental and theoretical investigations on pallet racks connections and proposed a component model. The experimental results were satisfactorily validated by the component model. Aguirre [8] studied the performance of SPR BCCs under both static and dynamic loadings. The results showed that the connection failure occurred due to deformation of the hooks in the beam end connector. Bajoria and Talikoti [9] tested SPR BCCs using both the cantilever and the double cantilever test methods. When compared with full frame test results, the double cantilever test showed better results than the simple cantilever test. Filiatrault et al. [10] tested the rotational stiffness and seismic response of various types of connector under low vibrations. Prabha et al. [11] determined the effects of the influencing parameters on the performance of SPR BCCs. The thickness of the column, depth of the beam and number of tabs in the beam end connector highly influenced the connection performance. Zhao et al. [12] investigated the performance of storage rack BCCs under flexure. Different constructional details were used and the agreement of results with the formulae provided in the relevant standards was discussed.

This study attempts to evaluate the improvement in SPR BCC performance under static loading by varying the most influential parameters. Thirty-two variant experiments were conducted on the pallet rack connections by varying the depth of beam, the thickness of the column and the number of tabs in the beam end connector. The experiments were carried out by means of the double cantilever test method. The major failure modes and both the moment-rotation (M-θ) and load-strain behavior of the connection were observed and the influence of the parameters on the overall connection performance were evaluated. A comparative study to calculate the connection stiffness using the three different methods like the initial stiffness method, slope to half-ultimate moment method and the equal area method was carried out.

## Experimental Investigations

### Material Properties

Cold formed steel sections were used for columns and beams. The beam end connectors were manufactured of hot rolled steel. The material properties of members and beam end connectors were obtained using the tensile coupon test and are given in Table 1.

**Table 1:** Material properties of specimens.

Member	Young’s Modulus ‘E’ (GPa)	Poisson’s ratio ‘v’	Yield Strength ‘fy’ (MPa)	Ultimate Strength ‘fu’ (MPa)
Column	210	0.3	459	575
Beam	210	0.3	353	497
Beam end connector	210	0.3	263	365

### Specimen Details

**Fig 1:**
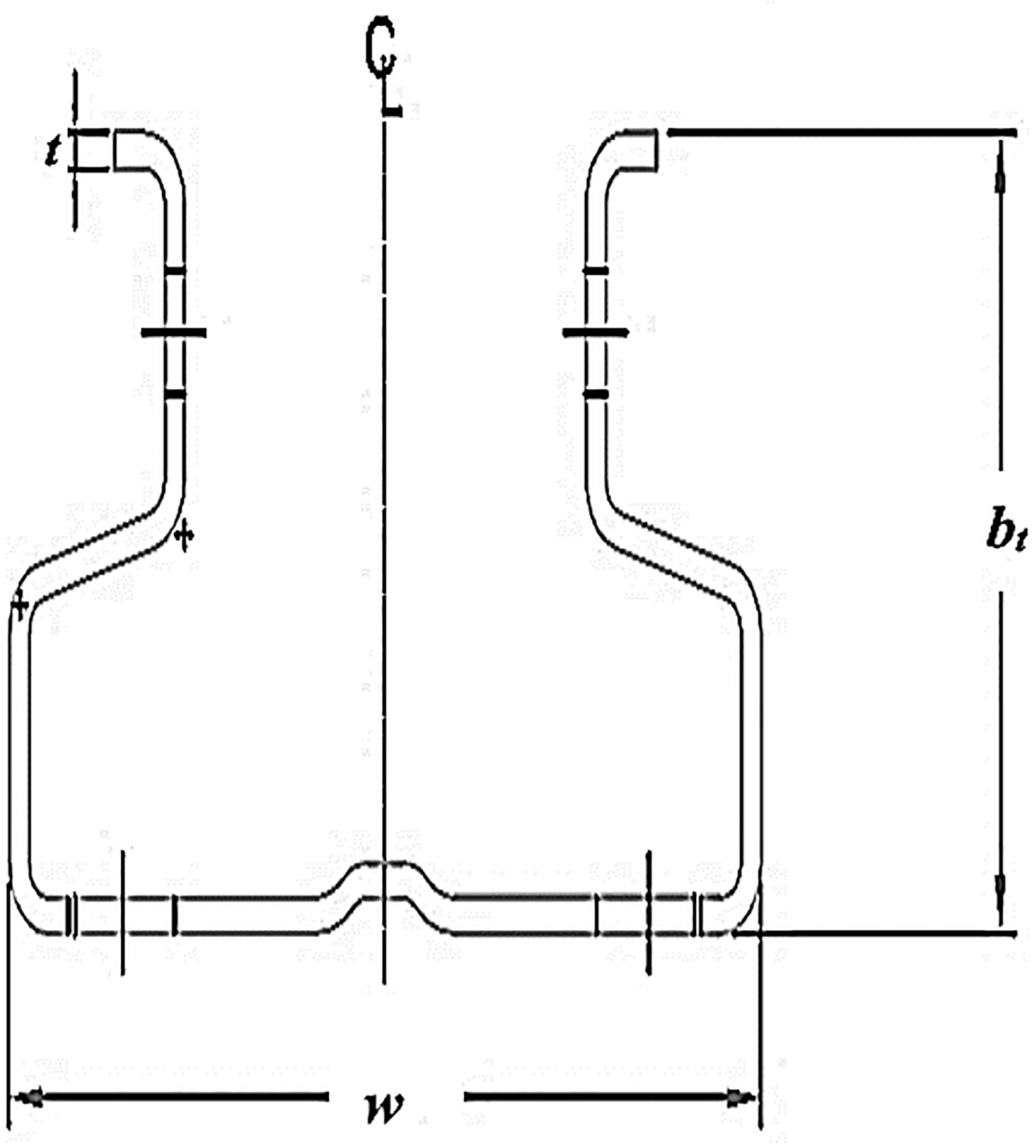
Cross-section of the column.

A total of 32 tests were carried out, composed of four trials of each set of specimens, which were distinguished by two different column thicknesses, four different beam depths and the number of tabs in the beam end connector being either four or five. The column specimens were distinguished by means of their thickness. Column ‘A’ had a thickness of 2.0 mm and column ‘B’ had a thickness of 2.6 mm. The height of the column was kept constant throughout the investigations and was limited to 500 mm. The cross-section of the column is illustrated in Fig 1. The details of the dimensions and section properties of the columns are given in Table 2.

**Table 2:** Dimension details and section properties of columns.

Column Thickness ‘t’ (mm)	2.0	2.6
Flange width ‘b_t_’ (mm)	67.6	68.3
Web ‘w’ (mm)	112.2	113.1
Height ‘h’ (mm)	500	500
Cross section area ‘A’ (cm^2^)	5.73	7.48

**Fig 2:**
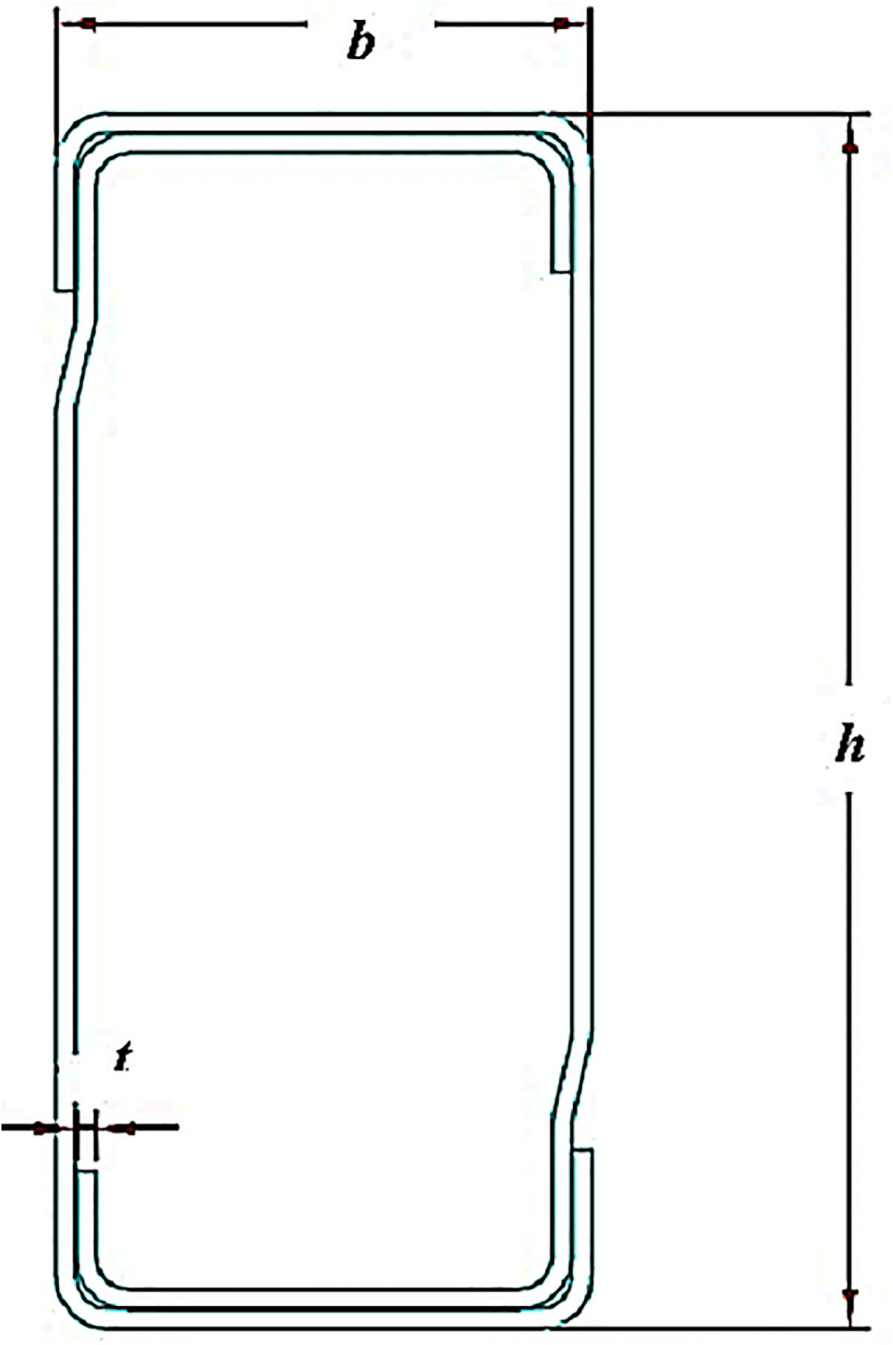
Cross-section of the beam.

Box-beams with four different depth values, namely B1, B2, B3 and B4 were used for experimental testing. Beams B1 and B2 had a four tab beam end connector, while for B3 and B4, the connector had five tabs. The cross section of the box-beam is represented in Fig 2. The dimensions and section properties of the beam sections are given in Table 3. All the dimensions of the specimens are the measured values.

**Table 3:** Dimension details and section properties of beams.

Type of beam	Width ‘b’ (mm)	Depth ‘h’ (mm)	Thickness ‘t’ (mm)	Cross-sectional Area ‘A’ (mm)	Center of Gravity ‘CG’ (mm) (x-x)	Center of Gravity ‘CG’ (mm) (y-y)	Moment of Inertia ‘M’ (cm) (x-x)	Moment of Inertia ‘M’ (cm) (y-y)	Section Modulus ‘S’ (cm) (x-x)	Section Modulus ‘S’ (cm) (y-y)
B1	40	92	1.5	387	20	46	42.197	11.5	9.173	5.75
B2	40	110	1.5	441	20	55	65.945	13.5	11.99	6.75
B3	50	125	1.5	516	25	62.5	102.6	24.6	16.415	9.862
B4	50	150	1.5	591	25	75	162.11	29.03	21.615	11.62

**Fig 3:**
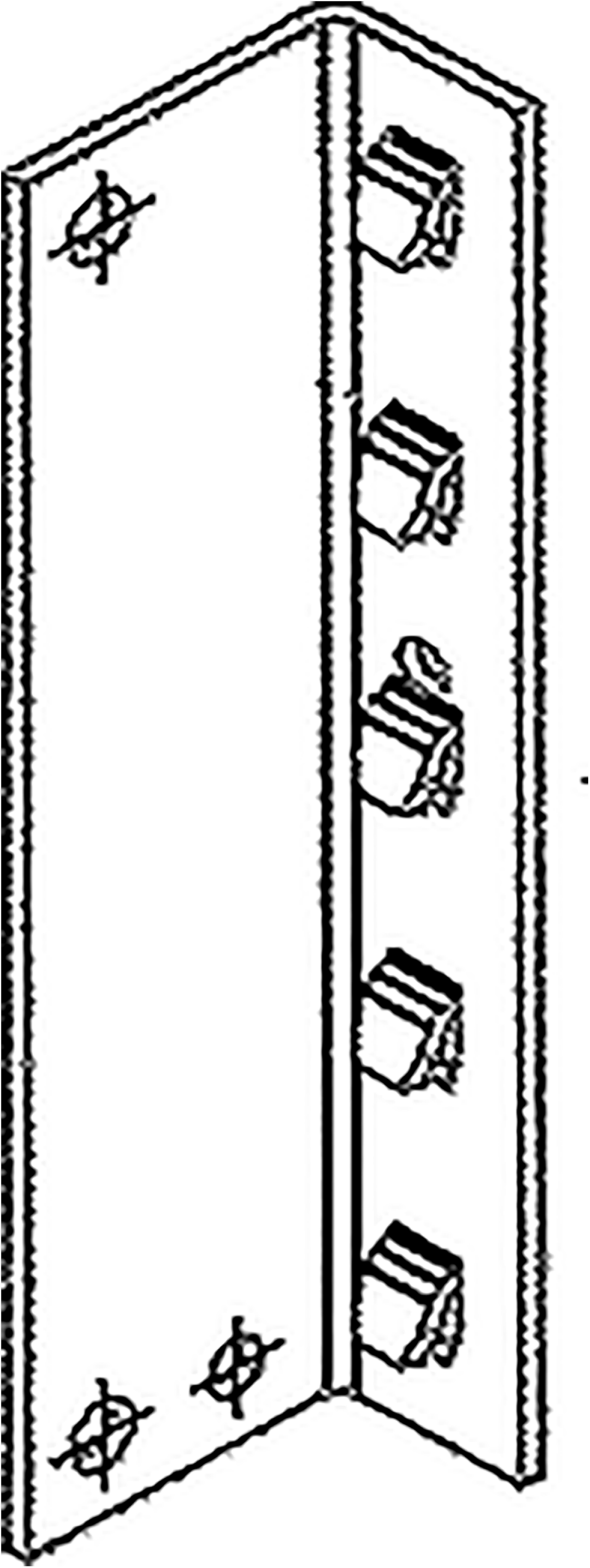
Cross-section of the beam end connector.

The geometry of the beam end connectors was distinguished by the number of tabs in the beam end connector. Connector ‘A’ had four tabs and connector ‘B’ had five tabs. The cross-section of the beam end connector is shown in Fig 3.

For a clarified representation of the groups of specimens under investigation, each set of experiments was given a specific specimen ID, which is listed in Table 4 along with the number of tests performed on each set of specimen. For example, in the specimen ID ‘2.0UT-92BD-4T’, 2.0UT represents the column thickness as being 2.0 mm, 92 BD represents the depth of beam as being 92 mm and 4T represent the number of tabs in the beam end connector, which is four.

**Table 4:** Details of specimens’ ID.

Specimen Set	Specimen ID	No. of tests performed
1	2.0UT-92BD-4T	4
2	2.0UT-110BD-4T	4
3	2.0UT-125BD-5T	4
4	2.0UT-150BD-5T	4
5	2.6UT-92BD-4T	4
6	2.6UT-110BD-4T	4
7	2.6UT-125BD-5T	4
8	2.6UT-150BD-5T	4

### Selection of Test Method

To investigate the behavior of the beam end connector, Rack Manufacturers Institute (RMI) [13] suggests alternative testing methods. These methods are the ‘portal frame testing method’ and ‘cantilever testing method’. However, the European Committee of Standardization (EN 15512) [14] only suggests the cantilever testing method.

The cantilever test can be further extended by attaching one more beam to the other side of the column, which accordingly, is called the double cantilever test method. Though, this test method has not been described in any of the standards yet, however, the literature has proven that when compared to full scale rack testing, the double cantilever test predicts the connection behavior more effectively than cantilever test method [9].

### Testing Arrangement

In this study, the double-cantilever test method was adopted to predict the M-θ behavior of the connection. Initially, the column was placed and aligned below the actuator. Two beams were connected at the center of the column on both left and right sides. The lateral movement of beams was restricted by restraining them by means of two rectangular hollow sections welded to the angle sections and bolted to the strong floor. The unconnected ends of the beams were supported by roller supports on the left and right sides. The effective distance between the roller supports was 2 m. The tabs of the beam end connectors were reversely hooked in the column perforations. A locking pin was used to avoid any change in the position of the column or the connector due to accidental uplift. A small amount of pre-load was applied initially and the displacement measuring devices were installed. When a tab is not properly engaged with the column, a larger initial rotation in the connection may occur; therefore, it is required to give an initial loading to make sure the beam end connector properly engages with the column. In contrast to the traditional test set-ups where the beam transmits the load to the connected column, the load was applied to the top of the column in a displacement control based method. The load was applied using a 50 kN hydraulic actuator controlled by the computer at a rate of 3 mm/min until the connection failure. The load was applied to the top of the column which caused compression in the top of the beam end connector and tension at the bottom.

**Fig 4:**
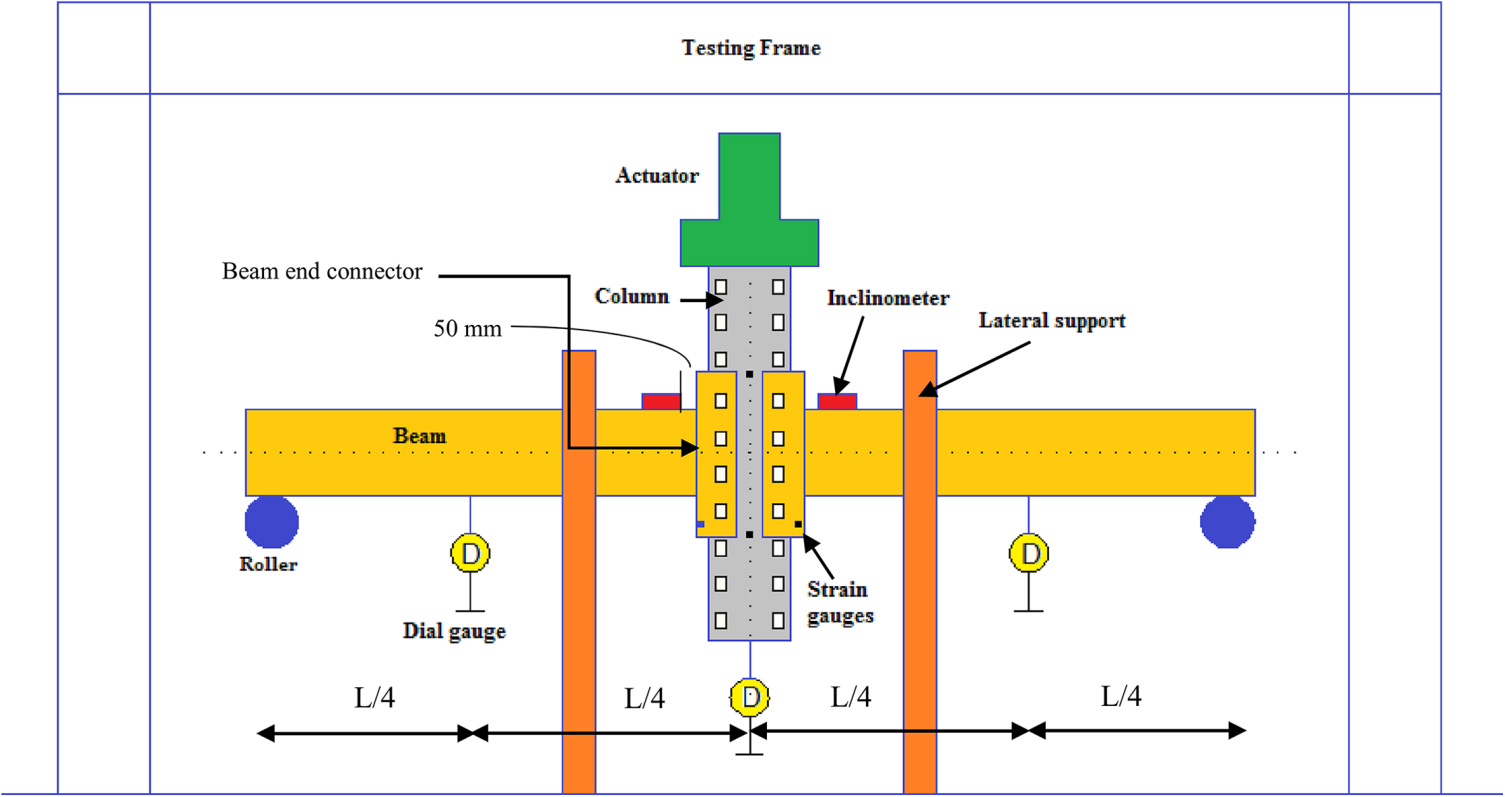
Schematic diagram of test set-up.

In order to achieve a set of information about the behavior of the connection throughout the entire range of applied loading, three different types of measurement were made in the tests besides loading. Strain readings were made to monitor the yielding of steel, displacements were measured to obtain the load-deflection behavior and rotation measurements were taken to obtain the M-θ characteristics. Two digital inclinometers were placed on the top flanges of the beams on either side at a distance of 50 mm from the face of the column to directly record the rotation of beams in degrees. Deflection measurements using linear variable differential transducers (LVDTs) having a measurement range from 50 mm–200 mm were also installed. In order to measure any deflection in beams due to the applied load, two LVDTs were installed at a distance of L/4 from the center of roller support, on the beams on either side. One LVDT was placed at the bottom of the column to observe central deflection. For the tests involving beams B1 and B2, three strain gauges were pasted. One strain gauge (S1) was pasted in the column web near the top surface of the beam end connector to estimate the tensile strain. The other two strain gauges (S3 and S4) were pasted near the bottom slot of the beam end connector in the tension region. For beams B3 and B4, four strain gauges were installed. Three strain gauges were pasted in the same position as for B1 and B2, whereas an additional strain gauge (S2) was installed in the lower portion of column web near the bottom surface of the beam end connector. The distance between the strain gauges S1 and S2 was equal to the depth of the beam end connector used for testing. Readings from the strain gauges and LVDTs were recorded onto the computer system through data logger. The schematic diagram of test set-up and the locations of instrumentation are presented in Fig 4.

## Results and Discussion

### Moment-Rotation (M-θ) Behavior

The moment was calculated by the following equation:

$$\text{Moment}\;(M)=\frac{P}{2}\times \langle \frac{L}{2}-\frac{w}{2}\rangle$$ (1)

L is the length between the supports, w/2 is the half width of the column; as the bending moment is to be calculated in the beam end connector, the half width of the column is subtracted from L/2.

**Fig 5:**
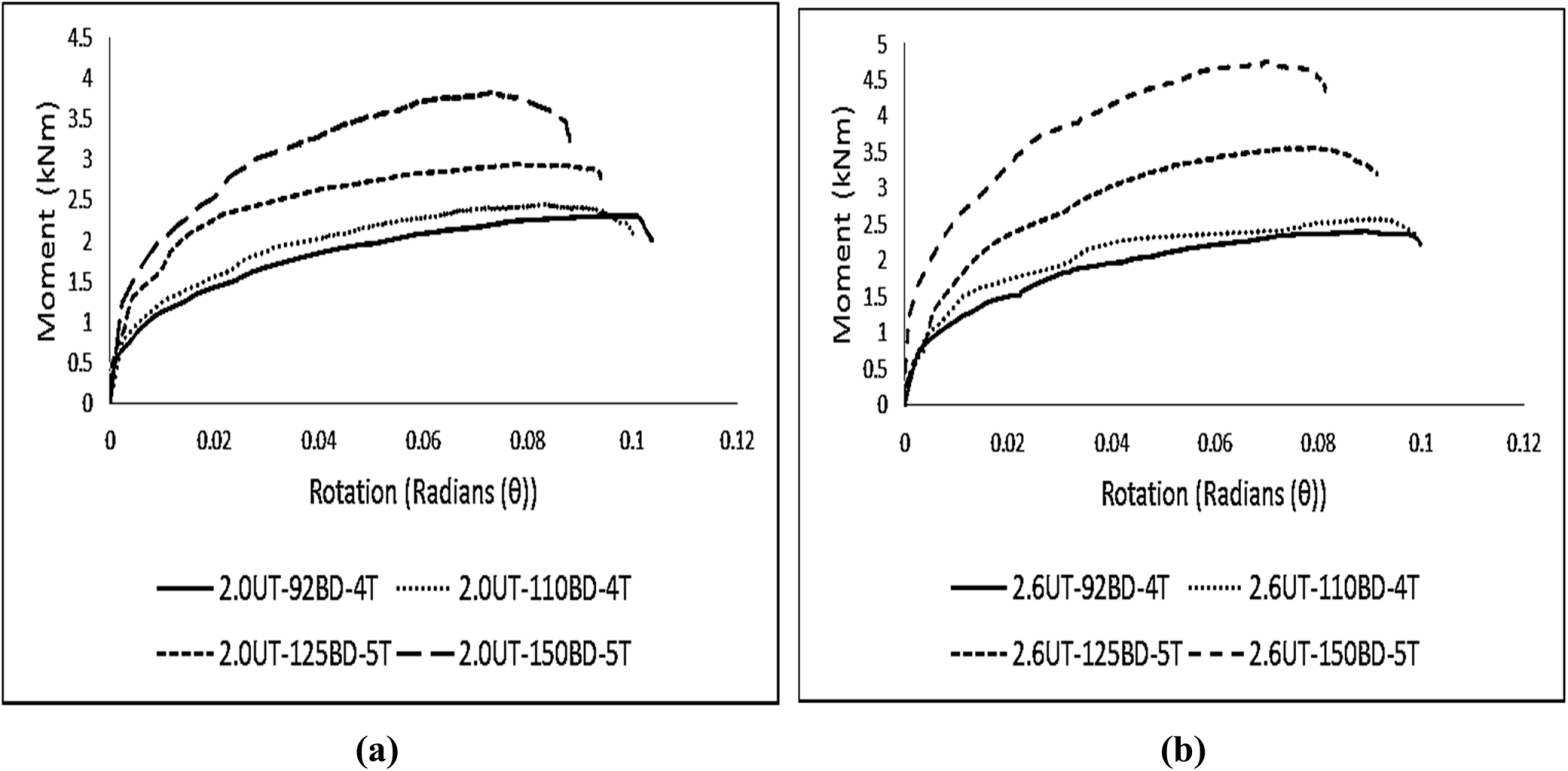
Average M-θ graphs for each set of specimens. **M-θ graphs for specimens with column A, (b) M-θ graphs for specimens with column B.** Contrary to an idealized graph of connections, these curves indicate non-linear behavior from the starting point. The major reasons for this overall non-linear behavior is due to the relative slippage between the column and the beam end connector, yielding of the tabs, or some points on the end-connector or the column perforation walls due to localized stress concentration, and geometrical non-linearity. The average results of experimental testing are given in Table 5.

In total, eight sets of specimens were tested. The average M-θ curves for each set of specimens are presented in Fig 5. For a clarified representation, the curves are divided into Fig 5(A) and 5(B) based on the difference in column thickness.

**Table 5:** Average test results.

Specimen	Failure Load (kN)	Ultimate Moment Capacity (kNm)	Rotation (Radians)
2.0UT-92BD-4T	4.89	2.31	0.10
2.0UT-110BD-4T	5.19	2.45	0.10
2.0UT-125BD-5T	6.27	2.96	0.094
2.0UT-150BD-5T	8.05	3.80	0.088
2.6UT-92BD-4T	5.17	2.44	0.10
2.6UT-110BD-4T	5.44	2.57	0.10
2.6UT-125BD-5T	7.54	3.56	0.091
2.6UT-150BD-5T	10.04	4.74	0.082

### Load-Strain Relationship

The load-strain behavior based on the applied load to the column and the strain recorded by the strain gauges was measured. In most of the tests, the strain gauges pasted in column showed that at the complete connection failure, the column experienced higher stress near the tension zone of the beam end connector. The S3 and S4 locations showed that, in majority of the tests, the welded joint for the beam and beam end connector promoted an uneven force distribution in the relatively shallow beams. Whereas, the specimens with larger beam depths, had a comparatively uniform force distribution. The behavior of the specimens with same column thickness and number of tabs in the beam end connector but different beam depths showed that the ratio of the beam depth to the depth of beam end connector in the beams with smaller depth was larger, which means that both the top and bottom tabs are closer to the top flange and bottom flange of the beam, respectively. In reality, the forces in the tabs were not evenly transferred to the beam, but transferred primarily to the portions of the flange and the outside web closer to the tab. The effect of this eccentricity was found to have considerable influence on the specimens with a smaller ratio between the beam depths to connector depths.

### Failure Modes

In this study, collectively, among all the experiments, three failure modes were observed: (i) tearing of the column material, (ii) yielding of the beam end connector, and (iii) fracture or yielding of the tabs. In most of the specimens, immediately after applying the load, a minor initial looseness of the beam end connectors was noticed due to the absence of bolts or welds in the connection, which induced lateral deformation in the specimens. As the loading continued, the gap between the connector’s surface and the column’s flange in the compression zone was closed and the space between the tension zone of the beam end connector and the column’s flange increased proportionally.

In the specimens 2.0UT-92BD-4T and 2.0UT-110BD-4T (relatively shallow beams and thin column), the pre-dominating failure mode was the failure of the tabs. Initially, the top tabs on both sides in compression zone (connected to the first slot of column making the connection) initially tried to tear the column web slots causing the drop in the load. However, a complete rupture of the top tab on both sides occurred before they distort the column web. The connection was able to sustain the load even after the complete rupture of top tabs. As the loading continued, the bottom two tabs in tension zone slit the column slots and came out by tearing the column flange and a considerable drop in load was observed. At this stage, connection failure was considered. The bottom two tabs were not completely ruptured, however, a noticeable deformation was observed. At failure, the beam end connector experienced a noticeable twist. This failure phenomenon was different in the case of specimens 2.0UT-125BD-5T and 2.0UT-150BD-5T. No complete rupture of top tabs was observed. The tabs in the tension zone were not deformed similar to the tabs in the specimens with relatively shallow beams. However, tearing of the column flange by bottom tabs on both sides was also observed in this case.

In the tests conducted on specimens 2.6UT-92BD-4T and 2.6UT-110BD-4T, the failure initiated due to the failure of tabs in both compression and tension zones. Increased column thickness removed the complete rupture of top tabs, however, the deformation of bottom tabs and the beam end connector was similar to the specimens 2.0UT-92BD-4T and 2.0UT-110BD-4T. The two tabs in the tension zone came out by tearing the column flange. When compared to the specimens 2.0UT-92BD-4T and 2.0UT-110BD-4T, the noticeable difference was the increased failure load which can be attributed to the increased column thickness. The distortion in column flange was minor in the case of specimens 2.6UT-125BD-5T and 2.6UT-150BD-5T. The last tabs in the tension side initially disengaged and finally came out of the column slots, completely. The deformation of the beam end connector was also not similar to the specimens with four tabs connector. No beam failure was observed in any specimen. It was noticed that an increase in the thickness of the column, made the tabs on the beam end connector experience larger deformation due to the in-plane moment.

**Fig 6:**
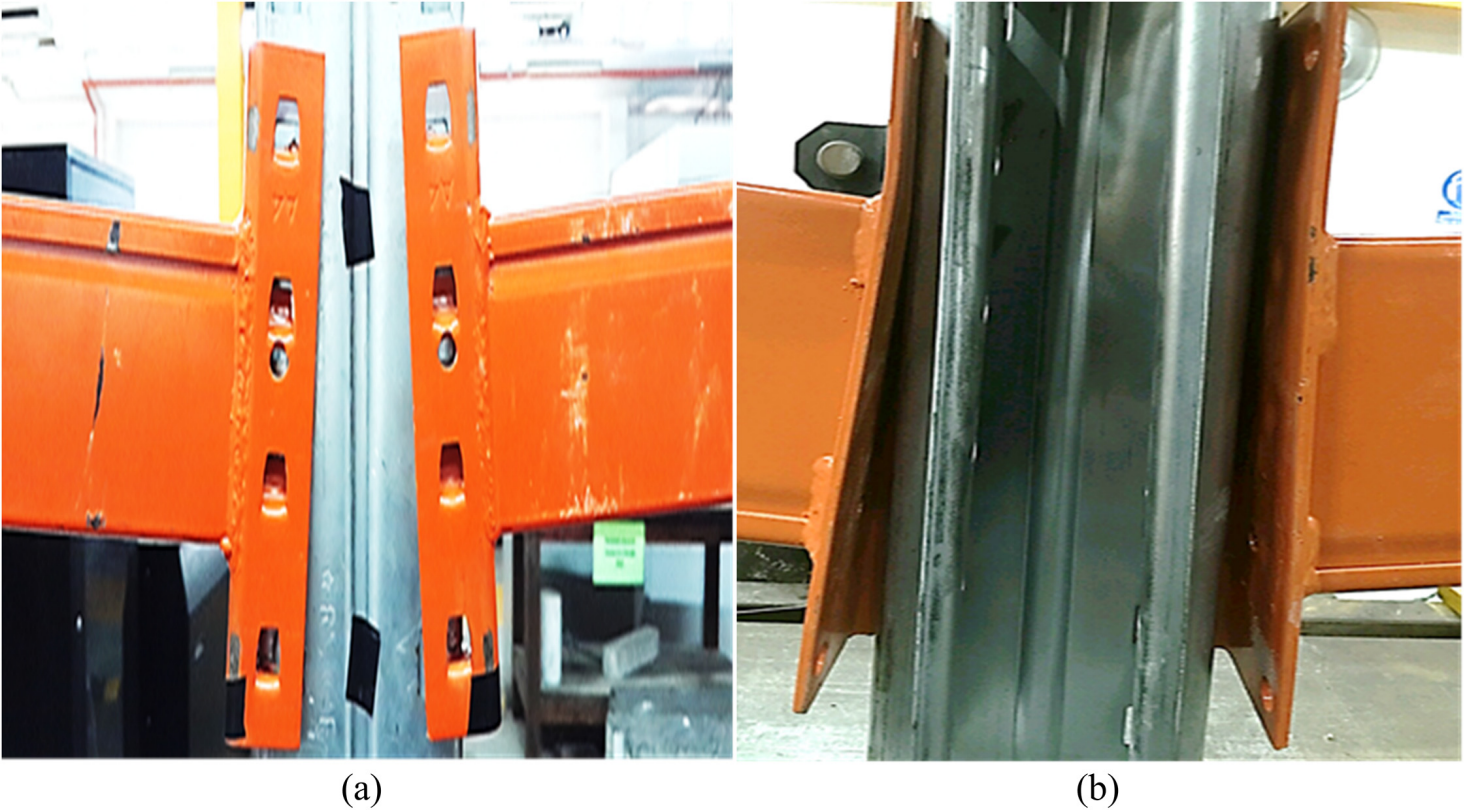
Connection failure. (a) Front view, (b) Back view.

**Fig 7:**
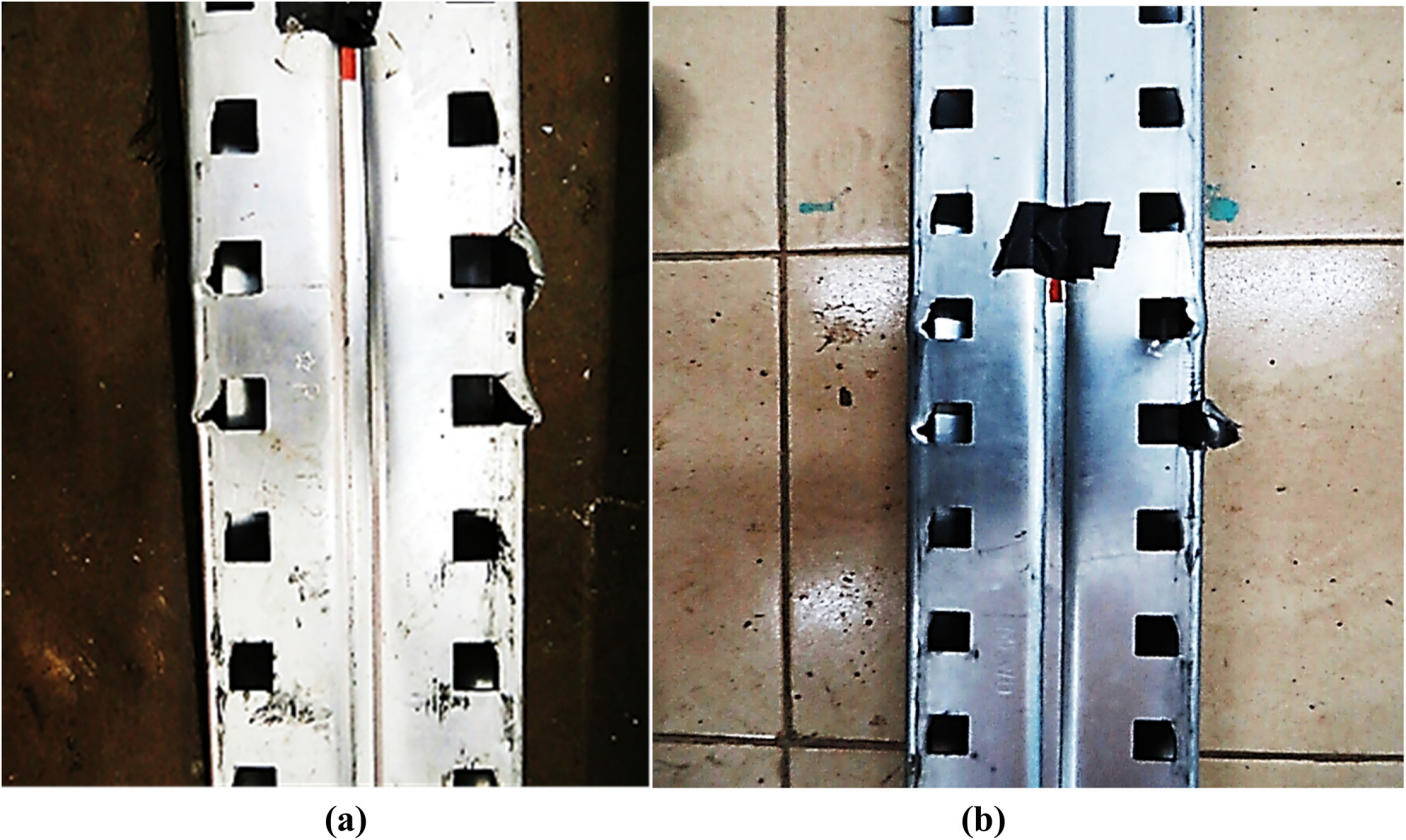
Deformation of columns. (a) Column A, (b) Column B.

**Fig 8:**
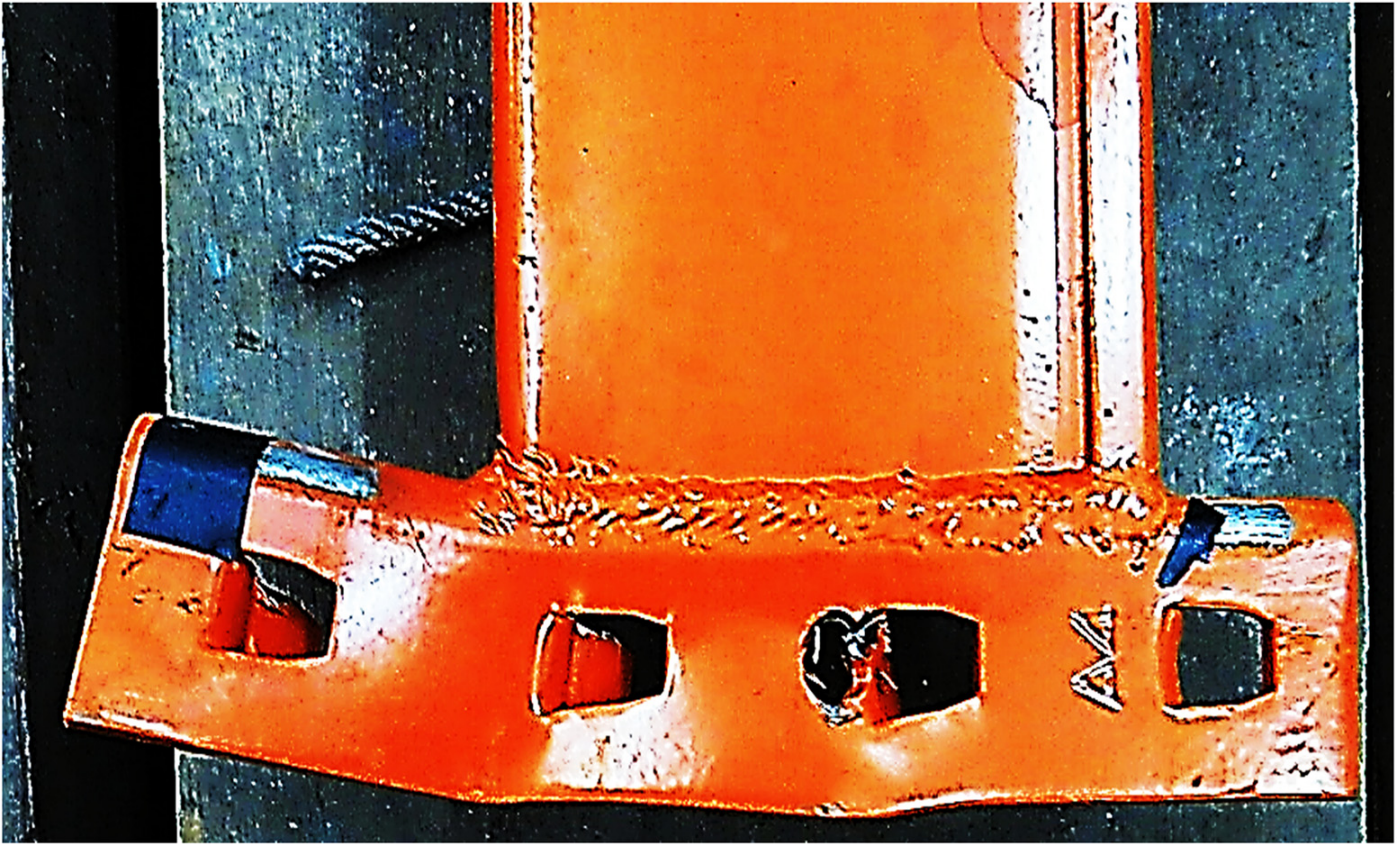
Deformation of the beam end connector.

**Fig 9:**
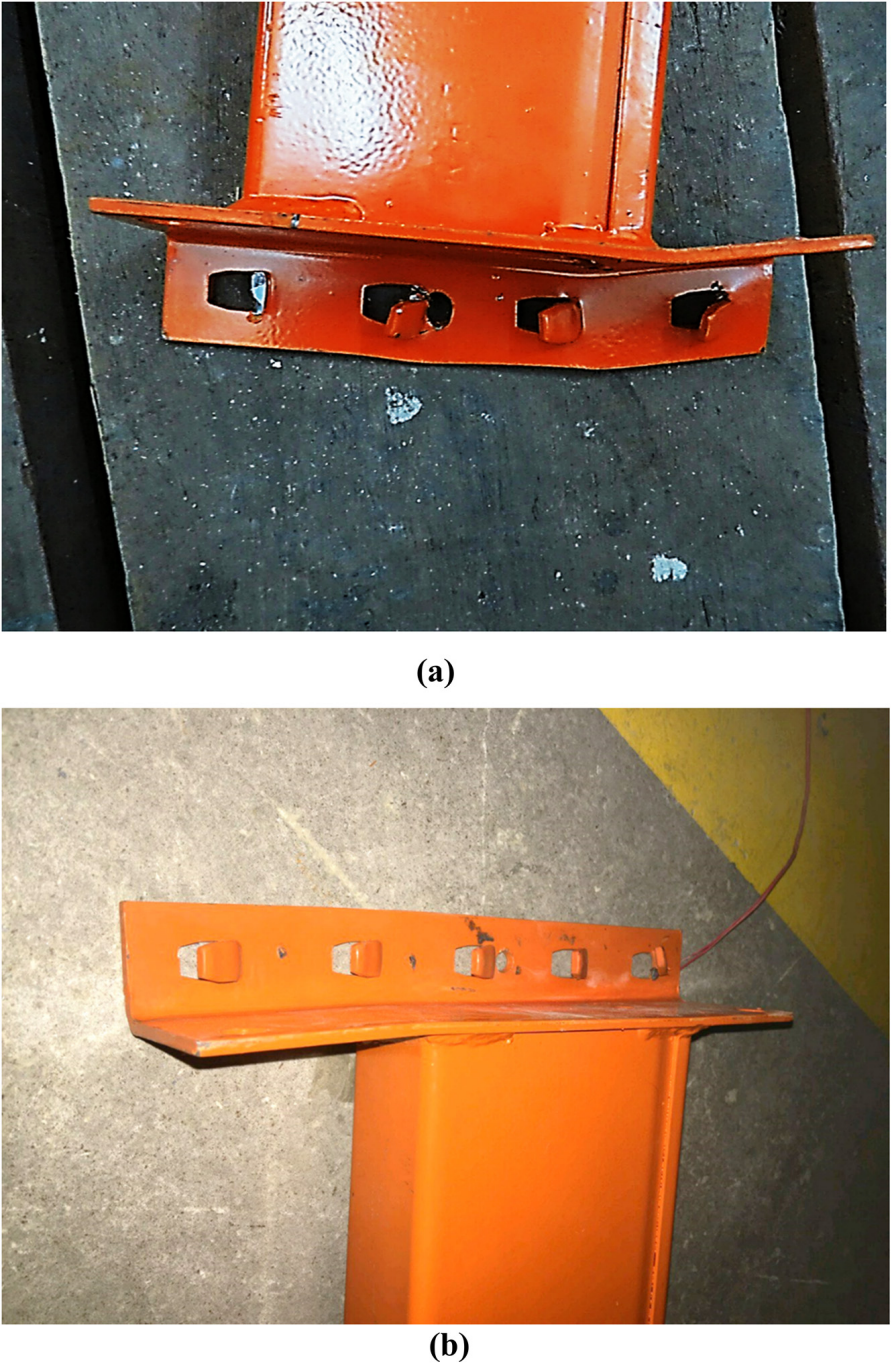
Deformation of tabs. (a) Deformation of tabs in connector ‘A’, (b) Deformation of tabs in connector ‘B’.

There was a variety in the types of failure among all connections. Only the maximum deformations observed during experiments are illustrated here. Fig 6(A) and 6(B) shows the front and back views of connection after failure, respectively. The deformation of columns A and B is illustrated in Fig 7(A) and 7(B), respectively. The deformation of the beam the connector is shown in Fig 8. The deformation of tabs in connectors A and B is illustrated in Fig 9(A) and 9(B), respectively.

### Stiffness Calculation

For designing a rack structure, the performance of the beam end connector is essential and thus the calculation methods require an accurate estimate of the beam end connector’s stiffness and strength. The criteria to calculate the stiffness of SPR BCCs is different in the current design codes. For the purpose of linear analysis, the RMI specification [13] suggest that the stiffness should be calculated as the slope of a line passing through the origin and a point on the M-θ curve at 85% of the maximum moment. EN 15512 [14] suggests that the rotational stiffness of the connector should be obtained as the slope of a line through the origin which isolates equal areas between it and the experimental curve, below the design moment corrected for yield and thickness. In addition, a variety of methods are adopted to measure the stiffness of the beam end connector. This study compares three different methodologies available in the literature [15] to calculate the connection stiffness. These methods are the initial stiffness, slope to half-ultimate moment and equal area methods that can be used to calculate the connection stiffness of any SPR BCC.

#### Initial Stiffness Method

In this method, the slope of the initial straight-line curve of the M-θ plot is measured, by imposing a best fit straight line. However, this method can lead to over-optimistic values if the characteristic is not linear for a substantial part of the range.

#### Slope to Half-Ultimate Moment Method

The slope to half-ultimate method suggests that the stiffness should be calculated by taking the slope of the line passing through the origin and the point at which the half of ultimate moment is reached. It eliminates the need to estimate the slope by fitting the straight line to the curve.

#### Equal Area Method

This method represents the true curve by an idealized characteristic comprising two straight lines, placed so that the work done to failure in the idealized case is the same as in the actual case. The rotational stiffness is taken as the slope of the line passing through the origin, which isolates equal areas between it and the experimental curve below the design moment.

#### Comparison of Methods to calculate connection stiffness

The stiffness for the tested specimens was evaluated using the initial stiffness method, slope to half-ultimate method and equal area method and is given in Table 6. The mean stiffness of the all four specimens in each set and the variance in the set data was calculated to predict the reliability of all three methods compared in this study. A small variance indicates that the data points tend to be very close to the mean (expected value) and hence to each other, while a high variance indicates that the data points are very spread out around the mean and from each other.

**Table 6:** Comparison of the Initial stiffness, Slope to half-ultimate moment and equal area methods.

Specimen set	Initial stiffness method			Slope to half-ultimate moment method			Equal area method		
	Stiffness of four specimens (kNm/rad)	Mean Stiffness (kNm/rad)	Variance	Stiffness of four specimens (kNm/rad)	Mean Stiffness (kNm/rad)	Variance	Stiffness of four specimens (kNm/rad)	Mean Stiffness (kNm/rad)	Variance
2.0UT-92BD-4T	79.02	72.81	67.48	55.65	60.78	210.12	31.7	32.3	0.27
2.0UT-92BD-4T	61.50	72.81	67.48	63.87	60.78	210.12	32.1	32.3	0.27
2.0UT-92BD-4T	71.97	72.81	67.48	79.02	60.78	210.12	32.9	32.3	0.27
2.0UT-92BD-4T	78.75	72.81	67.48	44.6	60.78	210.12	32.5	32.3	0.27
2.0UT-110BD-4T	82.75	83.92	8.73	70.80	70.71	13.68	35.3	36.2	3.08
2.0UT-110BD-4T	84.80	83.92	8.73	75.65	70.71	13.68	38.1	36.2	3.08
2.0UT-110BD-4T	80.60	83.92	8.73	66.78	70.71	13.68	34.3	36.2	3.08
2.0UT-110BD-4T	87.53	83.92	8.73	69.61	70.71	13.68	37.3	36.2	3.08
2.0UT-125BD-5T	90.86	89.13	48.03	79.80	77.21	22.90	57.82	54.29	5.57
2.0UT-125BD-5T	81.85	89.13	48.03	82.45	77.21	22.90	52.91	54.29	5.57
2.0UT-125BD-5T	85.89	89.13	48.03	71.90	77.21	22.90	53.08	54.29	5.57
2.0UT-125BD-5T	97.94	89.13	48.03	74.69	77.21	22.90	53.35	54.29	5.57
2.0UT-150BD-5T	101.32	110.7	167.13	89.12	89.90	38.44	79.40	79.21	13.48
2.0UT-150BD-5T	115.6	110.7	167.13	82.70	89.90	38.44	83.68	79.21	13.48
2.0UT-150BD-5T	126.7	110.7	167.13	97.83	89.90	38.44	79.17	79.21	13.48
2.0UT-150BD-5T	99.15	110.7	167.13	89.91	89.90	38.44	74.69	79.21	13.48
2.6UT-92BD-4T	85.50	73.60	63.85	42.60	36.67	33.41	31.23	35.9	11.97
2.6UT-92BD-4T	70.50	73.60	63.85	40.40	36.67	33.41	39.57	35.9	11.97
2.6UT-92BD-4T	68.30	73.60	63.85	30.34	36.67	33.41	36.7	35.9	11.97
2.6UT-92BD-4T	70.10	73.60	63.85	33.34	36.67	33.41	35.8	35.9	11.97
2.6UT-110BD-4T	76.14	85.10	38.86	88.60	77.21	59.50	39.2	42.8	7.79
2.6UT-110BD-4T	90.40	85.10	38.86	72.46	77.21	59.50	41.83	42.8	7.79
2.6UT-110BD-4T	87.80	85.10	38.86	72.45	77.21	59.50	45.03	42.8	7.79
2.6UT-110BD-4T	86.06	85.10	38.86	75.33	77.21	59.50	44.92	42.8	7.79
2.6UT-125BD-5T	78.60	82.85	536.91	88.60	79.97	51.69	70.72	69.54	27.60
2.6UT-125BD-5T	121.8	82.85	536.91	73.46	79.97	51.69	72.65	69.54	27.60
2.6UT-125BD-5T	131.1	82.85	536.91	74.67	79.97	51.69	73.20	69.54	27.60
2.6UT-125BD-5T	117.9	82.85	536.91	83.15	79.97	51.69	61.9	69.54	27.60
2.6UT-150BD-5T	147.9	143.66	430.16	114.6	120.47	142.14	101.4	97.86	68.70
2.6UT-150BD-5T	151.86	143.66	430.16	121.2	120.47	142.14	107.8	97.86	68.70
2.6UT-150BD-5T	161.2	143.66	430.16	136.8	120.47	142.14	89.8	97.86	68.70
2.6UT-150BD-5T	113.7	143.66	430.16	109.3	120.47	142.14	92.4	97.86	68.70

Table 6 shows that there is always an increase in the connection stiffness by increasing the thickness of column and number of tabs in the beam connectors for one particular type of column.

The analysis of the stiffness values obtained using the three methods shows that the initial stiffness method constantly gives an over-estimated value of stiffness. The slope to half-ultimate moment method showed high variance in the stiffness among the same size specimens in a given set of specimen. As compared to the initial stiffness and slope to half-ultimate moment methods; the equal area method has shown more consistent stiffness values among the specimens in each identified set. Moreover; based on the lowest variance in the set population as compared to the other two methods, the equal area method has provided relatively precise stiffness of the tested connections.

### Effect of parameters on connection performance

The effect of various parameters on the strength and stiffness of the tested connections is presented in this section. The reference stiffness is the stiffness obtained by the equal area method.

#### Effect of varying beam depth with constant column thickness and tabs in the connector

**Fig 10:**
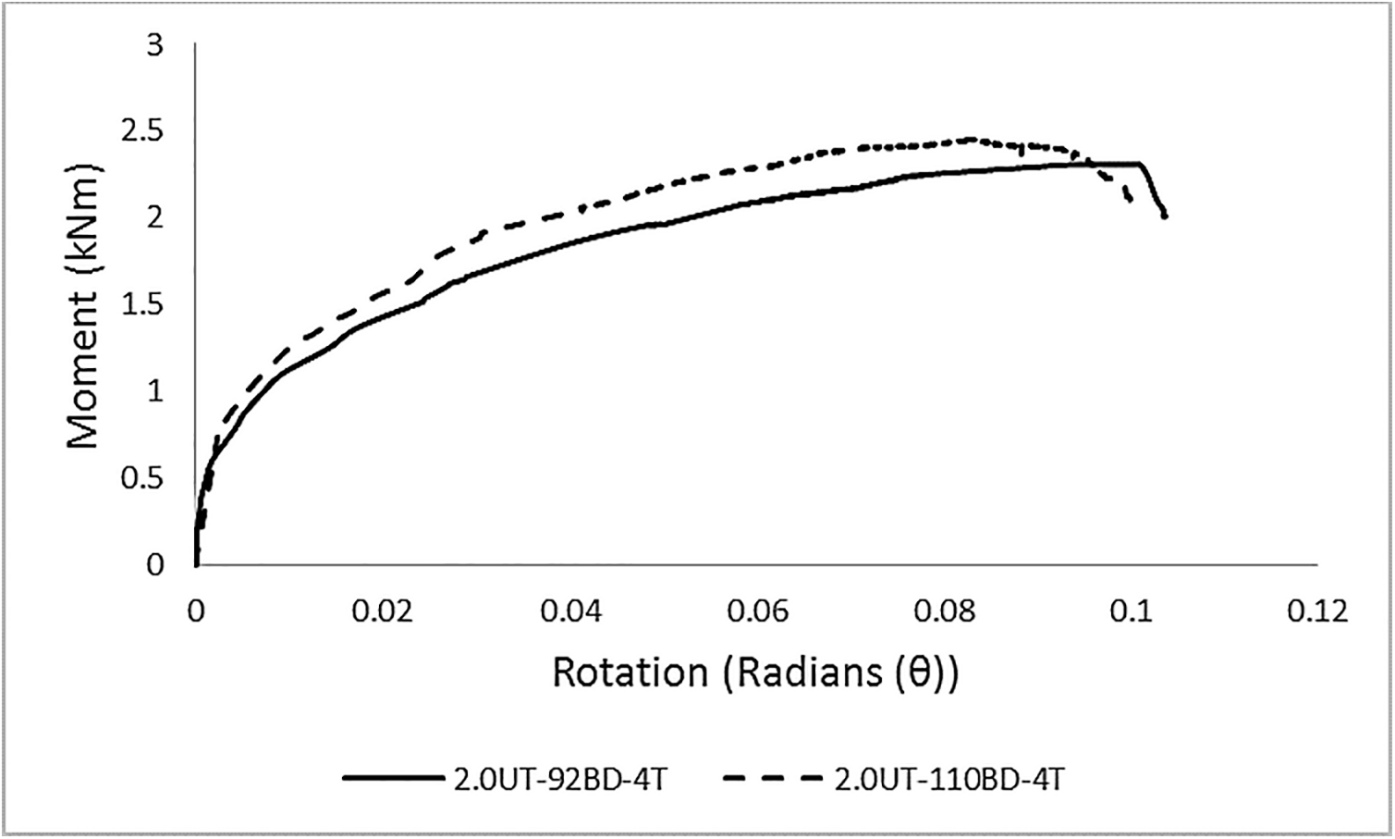
Effect of varying beam depth with constant column thickness and number of tabs in the beam end connector.

The rate of increase in the moment capacity of the connection by varying the depth of beams with same number of tabs in the beam end connectors was not much different for both 2.0 mm and 2.6 mm thick columns. In the case of connector A, for a constant column thickness of 2.0 mm, changing the beam depth from 92 mm showed a 6% increase in moment capacity for a depth of 110 mm. However, the stiffness was increased by 11%, as shown in Fig 10. The rate of percentage increase in the moment capacity and stiffness was almost similar for column B. Changing the beam depth from 92 mm to 110 mm for a constant column thickness of 2.6 mm, resulted in the increase in the moment capacity and stiffness by 6% and 16%, respectively, of the connection.

In the case of connector B, by keeping the thickness of the column constant at 2.0 mm, the effect of varying beam depth was considerable. The increased the beam depth enabled the connection to sustain higher moments. By keeping the column thickness constant at 2.0 mm, increasing beam depth from 125 mm to 150 mm increased the moment capacity and stiffness of the connection by 22% and 31%, respectively. Changing the beam depth from 125 mm to 150 mm for a constant column thickness of 2.6 mm, resulted in the increase in the moment capacity and stiffness by 24% and 29%, respectively, of the connection. This reveals that a higher increase in beam depth, increased the performance of the connection at a greater ratio. A progressive increase in the beam depth caused significant change in the moment capacity and stiffness.

#### Effect of column thickness on connection behavior

**Fig 11:**
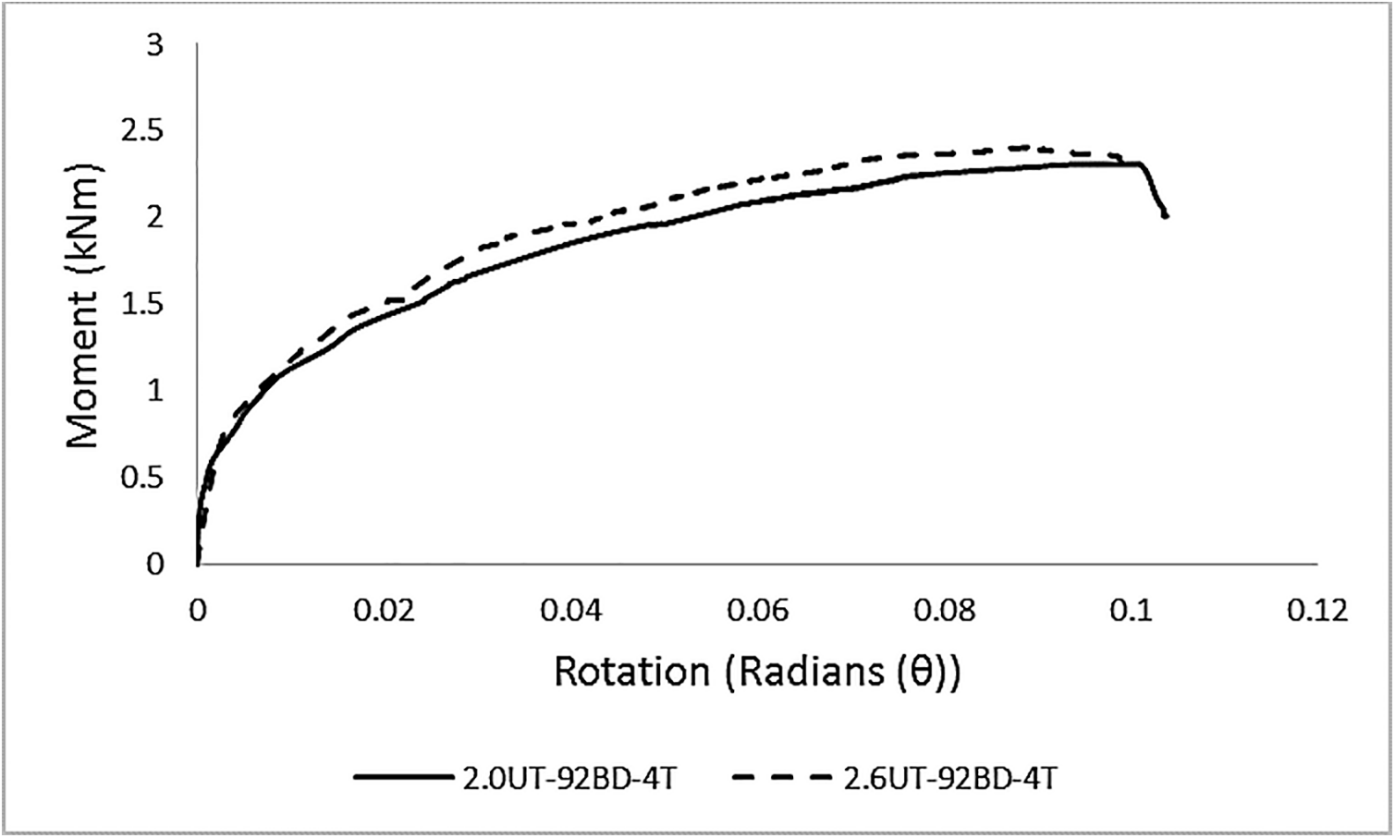
Effect of varying column thickness with constant beam depth and number of tabs in the beam end connector.

The resultant M-θ curve of connector ‘A’ with beam depth 92 mm and varying column thickness is presented in Fig 11. For B1, increasing the column thickness from 2.0 mm to 2.6 mm resulted in a marginal increase of 6% in the moment capacity of the connection. The stiffness was increased by 10%. For B2, increasing the column thickness from 2.0 mm to 2.6 mm resulted in an increase of 6% and 15% in the moment capacity and stiffness, respectively, indicating the influence of greater column thickness on connection performance.

#### Combined effect of variation in the geometry of beam end connector and beam depth

**Fig 12:**
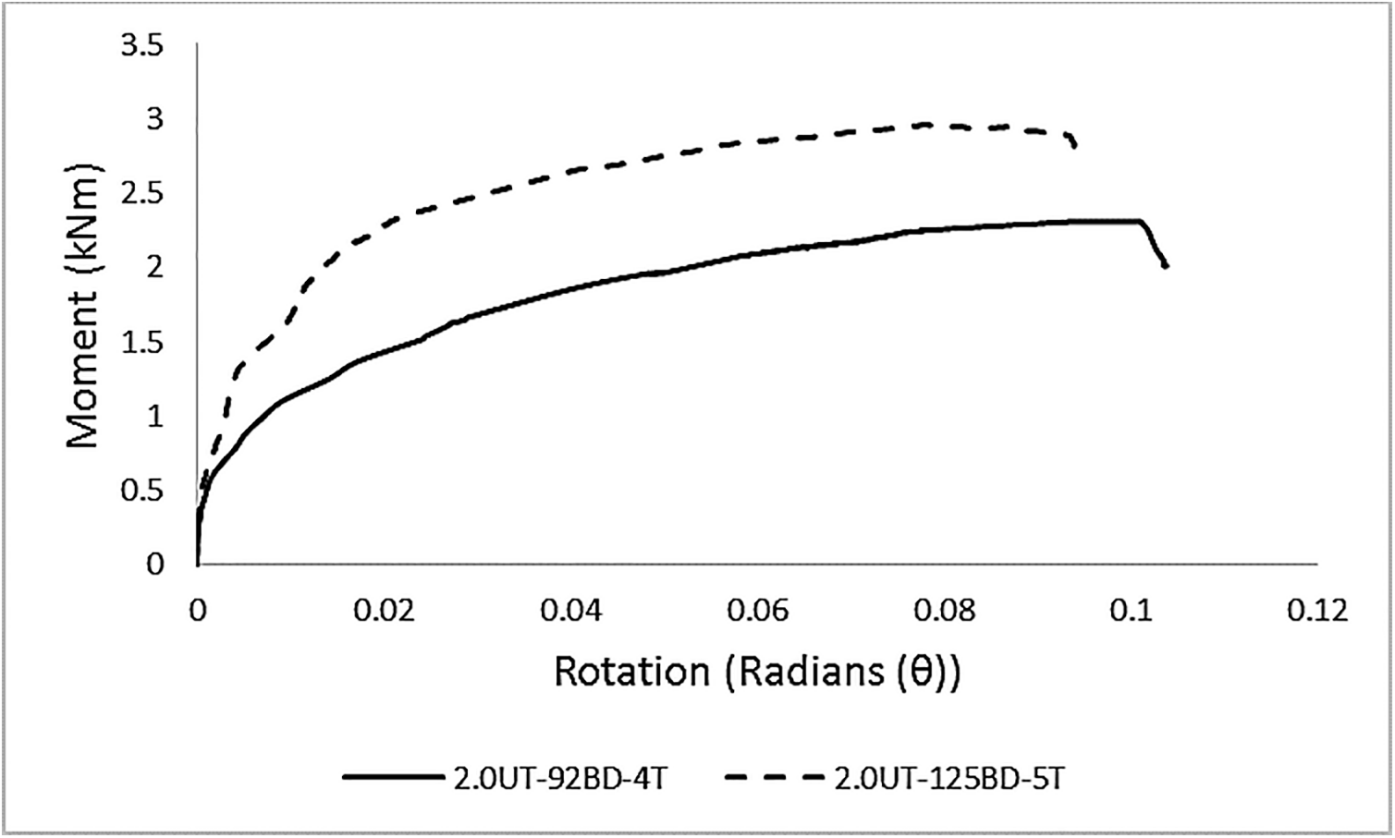
Effect of the geometry of the beam end connector.

During experimental investigations, the effect of number of tabs in the beam end connector was associated with the difference in beam depths. Therefore, the effect of connector geometry could be identified for the columns of same thickness only. For column ‘A’, changing the number of tabs from four to five (for increased beam depth from 92 mm to 125 mm) resulted in a 22% increase in the moment capacity and 40% in the stiffness of the connection, as shown in Fig 12. For column ‘B’, increasing the number of tabs from four to five (for increased beam depth from 92 mm to 125 mm) increased the moment capacity and stiffness of the connection by 31% and 48%, respectively. This demonstrated that increasing the number of connector tabs sufficiently enhanced the strength of the connection.

### Ductility

The ductility of a connection plays an important role in moment redistribution and is considered as a key parameter when the deformations are concentrated in the connection elements, as in the case of the BCCs tested in this study. The AISC [16] recommends that if the value of connection rotation at the maximum moment is ≥ 0.02 radians, the connection is considered as ductile, otherwise it should be considered as brittle. In the case of semi-rigid connections, the rotational capacity may effectively help in designing the connected beam. If the rotational capacity of a semi-rigid connection is sufficient to develop an effective hinge at mid-span of the connected beam, the beam can be designed plastically. In this study, at connection failure, the twist in the connector ‘B’ was considerably lesser than that observed for connector ‘A’. Table 5 shows changing the number of tabs from four to five along with a progressive increase in beam depth, showed a maximum difference of 12% in the rotational capacity of the connection at failure moment. By keeping the number of tabs and column thickness constant and considering the effect of beam depth on the connection performance, maximum 10% difference was observed in the rotational capacity at ultimate moment in the case of specimens 2.6UT-125BD-5T and 2.6UT-150BD-5T. An increase in column thickness did not show any considerable change on the maximum rotation of the connection. Collectively, all the connections tested in this study showed ductile behavior.

## Conclusion

This study attempted to evaluate the improvement in SPR BCCs’ performance under static loading by varying the most influential parameters. Thirty-two variant experiments were conducted on the pallet rack connections by varying the depth of beam, the thickness of the column and the number of tabs in the beam end connector. The experiments were carried out by means of the double cantilever test method. Both the M-θ and the load-strain behavior of the connection were observed and the influence of the parameters on the overall connection performance was tested.

The localized failure effect of SPR BCCs can be attributed to the wear and tear of the tool die. This is caused due to the repeated punching during the manufacturing of beam end connectors, which is the prime component in the connection. A slight variation in dimension in the component leads to a considerable variation in the value of connection strength and stiffness.

The following conclusions were made based on experimental investigations.

- The failure of the connection was initiated with the failure of the tabs. The tabs tried to tear the column web slot. A complete rupture of tabs was noticed in the case of specimen with relatively shallow beams and thin column. An increase in the number of tabs minimizes the deformation of the beam end connector. A combined effect of the higher number of tabs and greater beam depth is more influential as compared to varying the thickness of the column.
- The initial looseness of the connection gave rise to lateral deformation. Imperfections in the specimens tends to make the M-θ curve behave non-linearly even from a very early stage.
- Increased column thickness and greater beam depths enhanced the strength and stiffness of the connection. An increase in the thickness of the column, made the tabs on the beam end connector experience a larger deformation due to the in-plane moment.
